# Do not mistake symptomatic improvement for disease modification: therapeutic endpoints in secondary lower-extremity lymphedema require inflammation–fibrosis anchoring

**DOI:** 10.3389/fimmu.2026.1899048

**Published:** 2026-08-13

**Authors:** Jia-wen Wang, Li-Na Jian

**Affiliations:** 1 Department of Orthopedics, The Fourth Hospital of Hebei Medical University, Shijiazhuang, China; 2 Department of Pain Rehabilitation, The Fourth Hospital of Hebei Medical University, Shijiazhuang, China

**Keywords:** complete decongestive therapy, disease modification, fibrosis, immune–stromal remodeling, lower extremity, secondary lymphedema, therapeutic endpoints

## Abstract

Secondary lower-extremity lymphedema has long been regarded primarily as a disorder of impaired lymphatic fluid return. However, growing evidence indicates that its core pathology is a lymph stasis-driven immune–stromal remodeling process characterized by chronic inflammation, extracellular matrix remodeling, and progressive adipose deposition, which gradually reduces limb reversibility as the disease advances. Yet this mechanistic understanding remains disconnected from clinical therapeutic evaluation. Current endpoints still rely mainly on limb volume, circumference, and the degree of swelling reduction, which are insufficient to quantify fibrosis and adipose remodeling and cannot capture ongoing inflammatory and tissue progression when volume remains unchanged. This may create a misleading appearance of symptomatic improvement while the disease continues to progress toward a late fibroadipose stage, delaying timely referral for lymphaticovenular anastomosis (LVA) or vascularized lymph node transfer (VLNT) and increasing clinical risk. Therefore, we propose the introduction of Inflammation–Fibrosis–Anchored Endpoints (IFAE), which integrate four dimensions beyond volume-based assessment: immune–inflammatory activity, tissue remodeling, lymphatic function, and clinical events. Within this framework, traditional volume-based measures should be downgraded to surrogate indicators of the fluid dimension rather than treated as definitive markers of disease modification. Meanwhile, complete decongestive therapy (CDT) and adjunctive physical therapies should be repositioned as strategies for fluid–symptom management rather than disease-modifying interventions, to avoid equating symptomatic improvement with mechanistic disease control or cure.

## 1 Introduction

Secondary lower-extremity lymphedema has traditionally been viewed as a disorder caused by impaired lymphatic fluid return. However, recent studies indicate that this condition is closely linked to a lymph stasis-driven immune–stromal remodeling process characterized by progressive chronic inflammation, extracellular matrix remodeling, and adipose deposition (1). In this process, protein-rich interstitial stasis caused by lymphatic injury can trigger T cell-biased inflammation, fibroblast activation, and adipose expansion, thereby driving downstream cascades that contribute to the development of non-pitting limb changes and progressively reduce tissue reversibility (2).

Nevertheless, current clinical evaluation of therapeutic efficacy still relies mainly on limb volume, circumference measurement, bioimpedance spectroscopy, and patient-reported symptoms, while existing staging and consensus-based tools also emphasize volume- or fluid-based measurements (3, 4). These indicators can capture fluid distribution and overall fluid burden, but they are poorly suited to quantify tissue composition, fibrosis, and morphological abnormalities (5). More importantly, they cannot detect inflammatory modification when no measurable change occurs at the volume level. As a result, the disease may appear symptomatically improved or clinically stable while actually progressing toward a late fibroadipose stage, thereby delaying referral for lymphaticovenular anastomosis (LVA, a supermicrosurgical procedure that anastomoses lymphatic vessels to microvenules to drain lymph) or vascularized lymph node transfer (VLNT) (6). In advanced non-pitting lymphedema, excess limb volume is mainly composed of adipose tissue, which cannot be removed by decongestive therapy (7). Over time, persistent fibroadipose remodeling may further increase limb volume, leading some patients to show no improvement or even continued worsening during follow-up (8). In addition, studies have shown that decongestive therapy can significantly reduce circulating TNF-α without producing significant volume reduction, directly suggesting that inflammatory modification may occur independently of volume change (9). Similarly, after intensive decongestive therapy for lower-extremity lymphedema, reduced subcutaneous thickness may be accompanied by a significant increase in elastic modulus (10). Together, these findings suggest that, without improving lymphatic transport, conservative treatment may have limited effects on deep tissue remodeling and immune-barrier modification (11).

**Figure 1:**
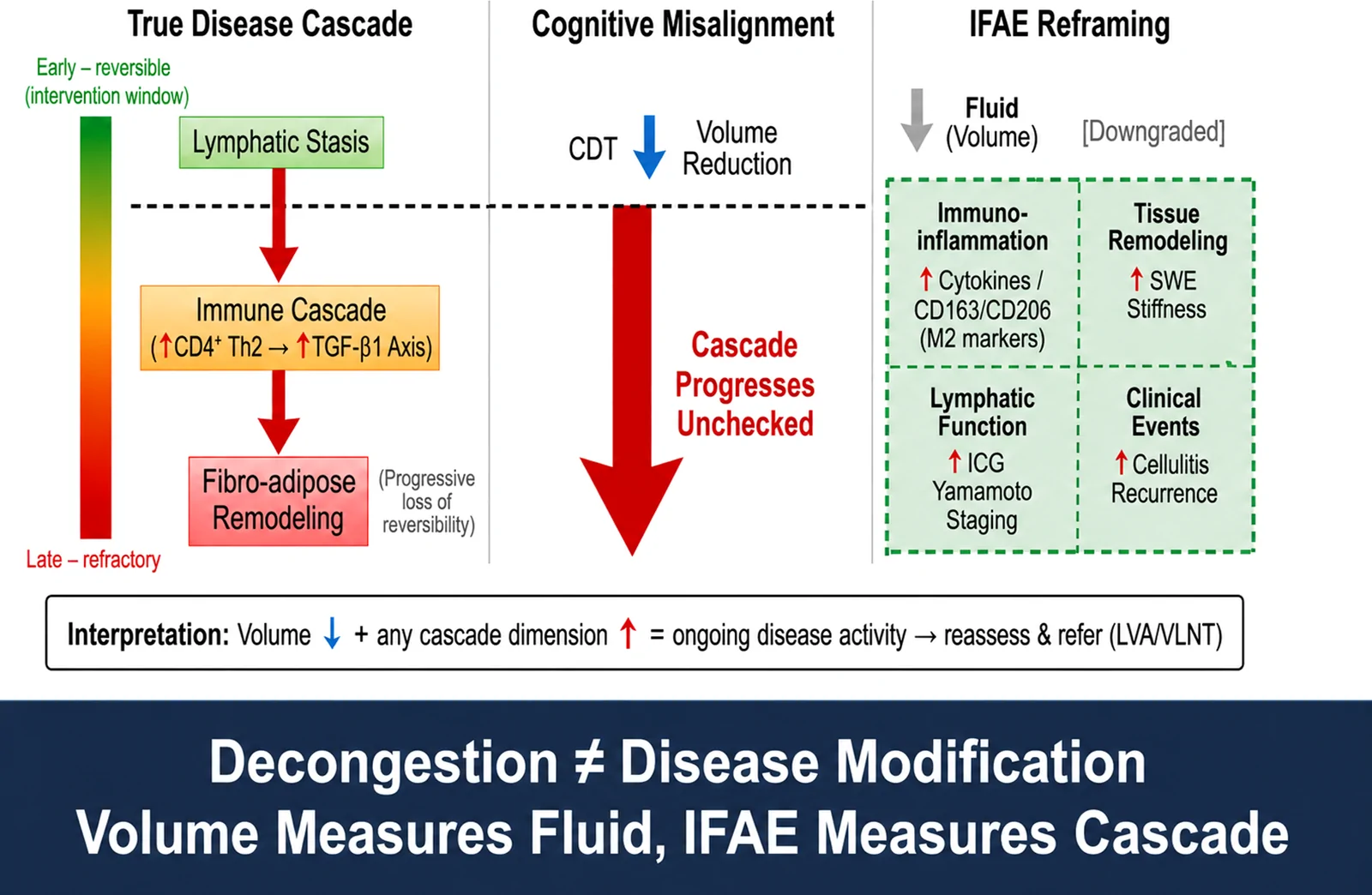
Decongestion is not disease modification: reframing therapeutic endpoints in lower-extremity lymphedema through Inflammation–Fibrosis–Anchored Endpoints (IFAE). The schematic contrasts the true pathological trajectory of lower-extremity secondary lymphedema with the level at which current efficacy evaluation operates, and illustrates the proposed corrective framework. Left (True Disease Cascade): following lymphatic stasis, a self-reinforcing immune–stromal cascade (CD4^+^ Th2 bias → TGF-β1 axis) drives fibro-adipose remodeling, with reversibility progressively declining as the disease advances. A vertical green-to-red gradient along the left margin encodes this progressive, unidirectional loss of reversibility: intervention within the early (green) window can still arrest the cascade, whereas the late (red) refractory stage is largely irreversible, which underscores why early, mechanism-directed intervention matters. Middle (Cognitive Misalignment): a horizontal dashed line separates the clinically visible fluid domain from the underlying immuno-stromal processes. Above the line, complete decongestive therapy (CDT) reduces limb volume; below it, a large red arrow indicates that the immune–fibrotic cascade continues to progress unchecked, so that volume reduction does not equate to interruption of the cascade. Right (IFAE Reframing): the proposed Inflammation–Fibrosis–Anchored Endpoints (IFAE) integrate four dimensions: immuno-inflammation (e.g., circulating cytokines, CD163/CD206, i.e., M2 macrophage markers), tissue remodeling (e.g., shear-wave elastography stiffness), lymphatic function (e.g., ICG Yamamoto staging), and clinical events (e.g., cellulitis recurrence), while limb volume is retained but downgraded to a proxy for the fluid dimension. Throughout, red upward arrows (↑) denote the direction of progression (rising CD4^+^ Th2, TGF-β1, cytokines, tissue stiffness, ICG stage, and cellulitis frequency), flagging the indicators that increase as disease advances. Listed indicators are representative examples rather than an exhaustive set. The interpretation key at the foot of the figure operationalizes the framework: a decrease in limb volume accompanied by worsening of any cascade-anchored dimension should be read as ongoing disease activity masked by symptomatic decongestion, prompting reassessment and timely referral for lymphaticovenular anastomosis (LVA) or vascularized lymph node transfer (VLNT). CDT, complete decongestive therapy; SWE, shear-wave elastography; ICG, indocyanine green; LVA, lymphaticovenular anastomosis; VLNT, vascularized lymph node transfer; IFAE, Inflammation–Fibrosis–Anchored Endpoints.

Therefore, we propose that therapeutic evaluation in lower-extremity lymphedema should move beyond a volume-centered and swelling-reduction-based framework by additionally incorporating Inflammation–Fibrosis–Anchored Endpoints (IFAE). In the absence of mechanistic endpoint evidence, CDT and adjunctive physical therapies should be repositioned as fluid–symptom management rather than disease-modifying interventions. In other words, symptomatic improvement should not be equated with mechanistic disease control or cure (**Figure 1**).

## 2 Mismatch between immune–stromal pathology and existing evaluation metrics

Secondary lower-extremity lymphedema has traditionally been regarded as a simple fluid-retention disorder. However, increasing evidence indicates that its core pathology also lies in lymph stasis-driven immune–stromal remodeling. In the early stage, pelvic lymph node dissection, external-beam radiotherapy, or recurrent infection may damage lymphatic vessels, leading to acute lymph accumulation and lower-limb swelling. Once the disease enters the chronic stage, however, its trajectory is largely determined by an immune–stromal cascade triggered by protein-rich interstitial stasis after lymphatic injury (12, 13). This cascade involves CD4^+^ T-cell activation, Th2 polarization, IL-4/IL-13 and TGF-β1 signaling, and fibroblast activation, thereby driving the transition from pitting edema to non-pitting fibroadipose remodeling and progressively reducing symptom reversibility. It also contributes to adipose deposition, skin sclerosis, recurrent cellulitis in the edematous region, and functional decline (14). Moreover, this process is influenced by independent risk factors such as the number of pelvic lymph nodes removed, external-beam radiotherapy, and previous episodes of cellulitis, suggesting that secondary lower-extremity lymphedema should not be viewed only through the lens of therapeutic measures and clinical complications. Rather, the self-amplifying rebound of immune–inflammatory events should be recognized as a central pathological concern (15, 16).

With regard to CD4^+^ T-cell activation, mouse tail and limb lymphatic ablation models have shown that CD4 deletion markedly attenuates the lymphedema phenotype, whereas adoptive transfer restores the phenotype and aggravates edema, supporting the causal role of CD4^+^ T cells in disease progression (17). At the human level, biopsy studies further corroborate this finding. Increased CD4^+^ infiltration and higher densities of IL-4/IL-13-expressing cells have been observed in lymphedematous tissues, with the degree of CD4^+^ infiltration showing a significant positive correlation with edema severity, thereby echoing and triangulating with the causal evidence from animal models. These findings indicate that CD4^+^ T-cell infiltration and activation are not merely bystander phenomena but key pathogenic drivers of lymphedema, with Th2-biased differentiation serving as a critical upstream process (18). This is followed by downstream fibrosis. TGF-β1 is significantly elevated in skin biopsies from patients with lymphedema and promotes skin sclerosis by activating fibroblasts, increasing extracellular matrix deposition, and enhancing fibroblast stiffness, thereby forming a positive feedback loop of “secretion–stiffening–further secretion”. Within this loop, progressively deposited extracellular matrix provides an essential structural foundation, and its continued evolution deepens skin sclerosis, ultimately generating non-pitting sclerotic tissue that is difficult to reverse through decongestive therapy alone (1, 19). Lymph fluid itself can also induce TGF-β-dependent epithelial–mesenchymal transition in primary keratinocytes and promote dermal fibrosis, making this a pathological process that is not reducible to fluid burden alone (20). In addition, adipose remodeling is also driven by non-canonical immune pathways. Tryptase-positive mast cells accumulating in lymphedematous tissues promote adipose fibrosis through the platelet-derived growth factor A pathway, thereby activating fibroblasts and preadipocytes, driving fibroadipose conversion, and constituting a non-canonical pathway independent of the classical CD4^+^/Th2–TGF-β1 axis (21). Together, these mechanisms form a multidimensional immune-mediated cascade that contributes to the refractory nature of lower-extremity lymphedema.

Although the 2023 International Society of Lymphology consensus suggests that the transition from late Stage II to Stage III is essentially an immune–stromal event, current therapeutic evaluation still relies heavily on limb circumference difference and volume difference. These measures are unable to quantify or capture immune-level changes, resulting in a systematic mismatch between pathological staging and clinical endpoints (3). This mismatch was reflected in a prospective cohort of 40 patients with secondary lymphedema who underwent lymphaticovenular anastomosis. After surgery, both excess volume percentage and the extracellular water/total body water ratio decreased significantly, whereas high-frequency ultrasound combined with shear-wave elastography showed increased subcutaneous tissue shear-wave velocity. This divergence between clinical and imaging features suggests that volume improvement and subcutaneous stiffness improvement may be dissociated (22). Shear-wave elastography has been validated for quantifying skin fibrosis and may serve as a biomarker of treatment response and prognosis; systematic reviews have also confirmed its ability to distinguish affected limbs from contralateral unaffected limbs (23, 24). This is not an isolated finding. CT-based quantitative follow-up of lower-extremity lymphedema after gynecological cancer surgery similarly showed that circumference changes and subcutaneous fibrosis changes are partially correlated but not fully concordant, indicating that circumference-based assessment alone may miss information on progression or improvement at the fibrotic layer (25). As a first-line treatment, CDT can reduce swelling and improve quality of life in the short term. However, its short-term decongestive benefit may conceal deeper fibrotic progression and delay referral for LVA or VLNT, particularly in refractory middle-to-late-stage disease characterized by skin thickening and adipose accumulation (22, 26). In addition, the current evidence for adjunctive physical therapies, such as short-wave diathermy and low-intensity pulsed ultrasound, remains largely limited to blood flow, temperature, or animal-model outcomes, with no direct human evidence demonstrating their ability to modify deep fibrosis (27, 28).

As fibroadipose remodeling progresses, recurrent cellulitis may also become more frequent. This is not simply a matter of skin-barrier disruption or bacterial colonization, but rather an integrated phenotype of impaired immune surveillance and lymphatic dysfunction. Lymph accumulation can promote M2 polarization of macrophages and weaken local antibacterial immunity, which may partly explain why recurrence rates remain high even with strict skin care (29). This suggests that cellulitis recurrence may serve as a hard endpoint reflecting impaired immune-barrier function, and its mechanism can be reasonably extrapolated to the lower extremities (30). At the same time, as a lymphatic functional dimension independent of fluid burden, worsening ICG dermal backflow staging and transitions in near-infrared fluorescence lymphatic imaging reflux patterns indicate further deterioration of lymphatic transport (31, 32). In addition, elevated circulating cytokine profiles suggest persistent immune activation even in the subclinical stage (33). Ultimately, when limb volume is stable or reduced but recurrence rates increase, ICG staging worsens, or circulating cytokines rise, the symptomatic appearance of “decongestion” does not demonstrate disease modification. Instead, it may obscure the true direction of disease progression and delay appropriate intervention (34). Viewed across the entire cascade, CD4^+^/Th2 signaling represents a causal driver in mouse models and is corroborated by human evidence, TGF-β1 and the mast cell/PDGF-A pathway act as causal drivers, and M2 polarization together with cellulitis recurrence function both as consequences and as feed-forward drivers, whereas limb volume is only a downstream marker. Therefore, subsequent assessment should appropriately downgrade volume and re-anchor therapeutic endpoints to causal and feed-forward nodes (1, 29, 30).

## 3 Discussion

We do not reject CDT or related therapeutic approaches. Rather, we emphasize that current clinical evaluation standards, which remain centered on limb volume and decongestion, are insufficiently aligned with immune–stromal pathological progression and with the mechanistic understanding reflected in international expert consensus. These standards therefore require refinement.

To address the disconnect between mechanism and assessment, we propose expanding therapeutic evaluation in lower-extremity lymphedema from a single volume-response framework to Inflammation–Fibrosis–Anchored Endpoints (IFAE). Within this framework, volume should be downgraded to a surrogate marker of the fluid dimension, while four additional dimensions should be incorporated: immune–inflammatory activity, tissue remodeling, lymphatic function, and clinical events (24, 30, 32, 33, 35). This approach would allow therapeutic evaluation to determine whether the immune–stromal cascade has truly been interrupted, rather than merely documenting improvement at the fluid level.

For follow-up assessment, we further recommend incorporating structural and functional monitoring into routine evaluation. Shear-wave elastography and high-frequency ultrasound may be used regularly to monitor tissue stiffness (22), while ICG lymphography and transitions in dermal backflow staging may help identify deterioration in lymphatic function and inform the timing of microsurgical intervention (34). In addition, dynamic monitoring of circulating cytokine profiles should be considered in high-risk patients, as specific cytokine patterns may help predict the onset of lymphedema (33). Together, these approaches may capture immune and mechanistic processes that traditional volume- or circumference-based indicators are unable to detect.

To facilitate the practical implementation of IFAE, we recommend a stratified framework supported by prospective validation. For stratification, although no single biomarker can currently serve independently as a disease-modifying endpoint, core reference standards may include shear-wave elastography-derived stiffness (22–24), ICG/Yamamoto staging (32), and cellulitis recurrence (16, 30). CD163/CD206 and other M2 macrophage markers may serve as mechanistic anchors (29), while circulating cytokine or chemokine profiles may provide molecular-level quantification (33, 35). Together, these elements may enable multidimensional stratification of IFAE. On this basis, the weight assigned to volume should be appropriately reduced, whereas cascade dimensions that more directly reflect immune–stromal progression—including increased tissue stiffness, ICG stage transition, cellulitis recurrence, and upward shifts in cytokine profiles—should be prioritized. This approach may quantify immune–stromal progression that conventional volume-based indicators cannot capture, reveal immune disease progression concealed beneath symptomatic decongestion, and improve the timing of referral, thereby reducing the risk of delayed intervention. However, the clinical value and relative contribution of the four dimensions are not equivalent: cellulitis recurrence is the most definitive and directly actionable clinical event; SWE is the most reliable bedside method for quantifying tissue sclerosis; ICG can reflect lymphatic function without being confounded by swelling; and circulating cytokines, although potentially the earliest warning signal, remain difficult to standardize in routine clinical practice. In addition, lymphedema shows substantial phenotypic heterogeneity across fluid-, fat-, and fibrosis-dominant states (36). Therefore, dimensional weights and parameters should not be fixed or uniform but dynamically adjusted according to the dominant phenotype. For example, structural readouts should receive greater weight in fibrosis-dominant disease, whereas clinical events should receive greater weight in patients with recurrent infection. This phenotype-guided weighting may more faithfully reflect the immune–stromal status of different individuals and disease states, while also providing a theoretical basis for subsequent empirical research.

The performance and final parameters of this framework should be determined through large-scale prospective cohort studies. Specifically, future studies should enroll a large number of patients with secondary lower-extremity lymphedema and define disease progression using multidimensional and multilayered clinically relevant indicators and outcomes that are independent of conventional volume-based assessment, including International Society of Lymphology stage transition, cellulitis recurrence, progression toward a non-pitting refractory stage, and eventual need for LVA or VLNT (3, 30). Time-dependent discrimination and reclassification metrics should also be used to test whether the cascade dimensions of IFAE outperform conventional volume-based indicators. Such studies may further determine which dimensions are mandatory, establish thresholds and weighting schemes, assess reproducibility, and provide an evaluation reference for future studies of lower-extremity lymphedema.

In addition, given the narrow endpoints and insufficient mechanistic evidence currently supporting many adjunctive physical therapies, as well as the clinical burden of recurrent cellulitis, CDT and adjunctive physical therapies should be defined as broad fluid–symptom management strategies rather than evidence-based disease-modifying interventions (3, 25, 26). Clinicians should remain alert to recurrent cellulitis or erysipelas, worsening ICG reflux staging, and prolonged failure to transition to LVA or VLNT when appropriate. In particular, when patients show no meaningful symptomatic improvement, timely microsurgical consultation should be considered rather than continuing CDT or other physical therapies alone, to avoid delaying effective intervention (22, 30).

## 4 Conclusion

The pathological understanding of secondary lower-extremity lymphedema has shifted from fluid retention alone to immune–stromal remodeling. However, current therapeutic evaluation remains largely focused on fluid burden, such as limb volume, and symptomatic outcomes, such as decongestion, making it difficult to capture the ongoing immune–fibrotic cascade. We therefore propose Inflammation–Fibrosis–Anchored Endpoints (IFAE), which integrate immune–inflammatory activity, tissue remodeling, lymphatic function, and clinical events beyond volume-based measures. This framework may improve therapeutic assessment by aligning evaluation with the immune–stromal mechanisms that drive disease progression, rather than limiting it to symptomatic improvement.

## Data Availability

The original contributions presented in the study are included in the article/supplementary material, further inquiries can be directed to the corresponding author/s.
